# Food Containing Bioactive Flavonoids and Other Phenolic or Sulfur Phytochemicals With Antiviral Effect: Can We Design a Promising Diet Against COVID-19?

**DOI:** 10.3389/fnut.2021.661331

**Published:** 2021-06-17

**Authors:** Martina Ghidoli, Federico Colombo, Stefano Sangiorgio, Michela Landoni, Luca Giupponi, Erik Nielsen, Roberto Pilu

**Affiliations:** ^1^Department of Agricultural and Environmental Sciences - Production Landscape, Agroenergy, Università degli Studi di Milano, Milan, Italy; ^2^Department of Bioscience, Università degli Studi di Milano, Milan, Italy; ^3^Centre of Applied Studies for the Sustainable Management and Protection of Mountain Areas – CRC Ge.S.Di.Mont., Università degli Studi di Milano, Edolo, Italy; ^4^Department of Biology and Biotechnology Università degli Studi di Pavia, Pavia, Italy

**Keywords:** flavonol, flavanone, phytochemicals, functional foods, COVID-19, diet

## Abstract

Since in late 2019, when the coronavirus 2 (SARS-CoV-2) pathogen of coronavirus disease 2019 (COVID-19) started to spread all over the world, causing the awful global pandemic we are still experiencing, an impressive number of biologists, infectious disease scientists, virologists, pharmacologists, molecular biologists, immunologists, and other researchers working in laboratories of all the advanced countries focused their research on the setting up of biotechnological tools, namely vaccines and monoclonal antibodies, as well as of rational design of drugs for therapeutic approaches. While vaccines have been quickly obtained, no satisfactory anti-Covid-19 preventive, or therapeutic approach has so far been discovered and approved. However, among the possible ways to achieve the goal of COVID-19 prevention or mitigation, there is one route, i.e., the diet, which until now has had little consideration. In fact, in the edible parts of plants supplying our food, there are a fair number of secondary metabolites mainly belonging to the large class of the flavonoids, endowed with antiviral or other health beneficial activities such as immunostimulating or anti-inflammatory action that could play a role in contributing to some extent to prevent or alleviate the viral infection and/or counteract the development of SARS induced by the novel coronavirus. In this review, a number of bioactive phytochemicals, in particular flavonoids, proven to be capable of providing some degree of protection against COVID-19, are browsed, illustrating their beneficial properties and mechanisms of action as well as their distribution in cultivated plant species which supply food for the human diet. Furthermore, room is also given to information regarding the amount in food, the resistance to cooking processes and, as a very important feature, the degree of bioavailability of these compounds. Concluding, remarks and perspectives for future studies aimed at increasing and improving knowledge and the possibility of using this natural complementary therapy to counteract COVID-19 and other viral pathologies are discussed.

## Introduction

**Figure 1:**
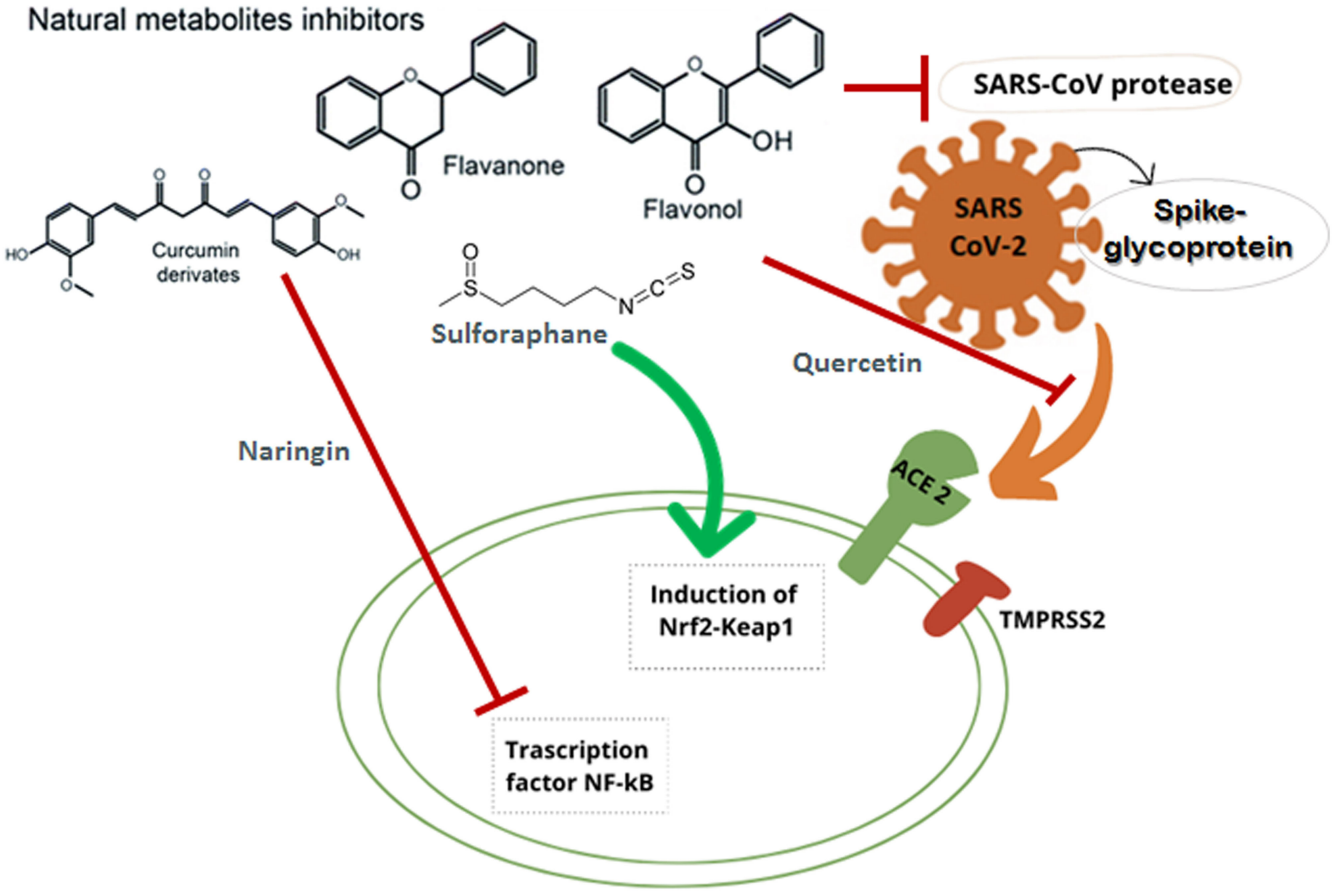
Schematic representation of the main mechanisms of action against SARS CoV-2 of different secondary plant metabolites: flavanones, flavonols, aromatic compounds, and sulfur compounds.

At the end of 2019, the World Health Organization (WHO) reported numerous cases of low respiratory tract infections in Wuhan (Hubei province, China) caused by a novel virus. The novel virus is a member of the *Coronaviridae* family and it was identified as Severe Acute Respiratory Syndrome Coronavirus 2 (SARS-CoV-2) due to its high similarity with another previously isolated coronavirus, SARS-CoV (1–4). Due to its spread all over the world, in March 2020, the WHO declared a pandemic (5). Over the recent decades, an increase in diseases caused by new coronaviruses has been reported in humans and animals (6). Among these, SARS-CoV (2002–2003) and MERS-CoV (2012) caused serious health problems and demonstrated the lethality of coronaviruses if they cross the species barrier and subsequently infect humans (7, 8). Currently, the SARS-CoV-2 generated the unprecedented COVID-19 (Coronavirus Disease of 2019) outbreak. This pathogen can affect several tissues at multiple levels in humans: from the cells of nose and throat down to the lung, and also invading the kidneys and the nervous system, where it can lead to severe illness and death (9, 10). The subjects at highest risk of developing severe COVID-19 symptoms are the elderly and those with major chronic diseases, such as diabetes, cancer and hypertension (11). MERS-CoV binds to dipeptidyl-peptidase 4 (DPP4) receptors to infect human cells (6), while both the original SARS-CoV and the novel virus SARS-CoV-2 bind to the angiotensin-converting enzyme 2 (ACE2). However, SARS-CoV-2 has a greater binding affinity to ACE2, presenting an higher infectivity compared to the previous SARS-CoV (12–16). Similarly to SARS-CoV, also in SARS-CoV-2, two open-reading-frames (ORF1a and ORF1b) are translated into two viral enzymes fundamental for virus replication: 3C-like protease (3CLpro) and papain-like protease (PLpro) (17). In this context, an essential role in the infection is played by the spike glycoprotein (S), located on the viral phospholipidic membrane surface (Figure 1). In particular, the receptor-binding domain (RBD) of the spike protein of SARS-CoV-2 binds strongly to ACE2 receptors after the activation by two host serine proteases (TMPRSS2 and furin). The entry of the virus into host cells causes an increase of the natural inflammatory response (defined as a cytokine storm), leading to serious problems particularly in the respiratory tract.

In synergy with therapeutic treatments and vaccines, we propose that the diet might play a significant role to prevent or to mitigate the symptoms of this illness. In fact, it is known that many phytochemicals have great potential in preventing viral infection, modulating immune responses, and decreasing the inflammatory response (18, 19). These natural molecules are present not only in a few medicinal plants, but also in many edible parts (seeds, fruits and vegetables) of cultivated plants which form part of the human diet. Consequently, we have available various “functional foods” that could complement our daily diet, with positive effects on both prevention and reduction of the severity of COVID-19 symptoms (Table 1).

**Table 1:** Main foods rich in bioactive molecules and their effectiveness against CoVs.

**Bioactive plant molecules**	**Compound**	**Main Source**	**Food Concentration (mg/100 g)**	**Effectiveness Against CoVs**	**References**
Flavonoids	Quercetin	Capers Buckwheat Onions	Capers (raw) 234 Buckwheat 184–535 Onions 120	SARS-CoV-2 proteases (3CLpro, PLpro), ACE2 receptor, glycoprotein-RBD Spike	(20–23)
	Kaempferol	Capers Saffron Brassicaceae	Capers (raw) 259 Saffron 205 Brassicaceae 30–60	SARS-CoV-2 protease, glycoprotein-RBD Spike, NF-kB	(24, 25)
	Naringenin	Citrus fruits Tomatoes	Grapefruit 53 Orange 11 Tangerine 11 Tomato 5–12	SARS-CoV-2 protease (3CLpro), ACE2 receptor, NF-kB	(26–28)
	Hesperetin	Citrus fruits	Orange 20–60 Tangerines 8–46 Lemon 4–41 Grapefruit 2–17	SARS-CoV-2 protease (3CLpro), glycoprotein-RBD Spike, ACE2 receptor	(29)
Other aromatic compounds	Curcumin	Turmeric	3,000	SARS-CoV-2 protease (3CLpro), glycoprotein-RBD Spike, ACE2 receptor	(30, 31)
	Phloretin	Apple Kumquat Pear Strawberry	40	Activation Nrf2 pathway, epigenetic regulation	(30, 32, 33)
	Epigallocatechin gallate (EGCG)	Tea	Green tea 7,380 White tea 4,245 Black tea 936	ACE2 receptor, NF-kB, epigenetic regulation	(34, 35)
Sulfur compounds	Sulforaphane	Brassicaceae	Broccoli 1,400	Activation Nrf2 pathway, epigenetic regulation	(36, 37)

Several foods, particularly fruits and vegetables, are rich in different natural compounds with beneficial effects on human health. In particular, various aromatic and a few sulfur compounds are known for their key roles as antioxidants, antivirals and anti-inflammatories (38). These bioactive phytochemicals may thus alleviate SARS-CoV-2 symptoms, decreasing the inflammatory responses (39, 40).

In this review, we present many natural plant-derived compounds whose intake can be implemented in the human diet and illustrate their antiviral potential or beneficial properties which may counteract COVID-19 progression. Furthermore, within the same species, the varieties characterized by a higher content of these phytochemicals are described. In particular, the focus is on the flavonoids flavonones and flavonols which are reported to be able to significantly counteract coronavirus infection and thus may also play a central role in protection against the novel COVID-19.

## Flavonoids

Flavonoids are secondary metabolites synthesized by plants. They are divided into different classes: anthocyanins, flavanols, dihydroflavonols, flavanones, flavones, flavonols, isoflavonoids, chalcones, and dihydrocalcones (18).

The maize flavonoid biosynthesis involves over 20 loci, and historically was the first elucidated plant metabolic pathway, due to the facility with which it enabled work with non-lethal mutants, and corn revealed itself to be the ideal model plant for a variety of different genetic studies.

**Figure 2:**
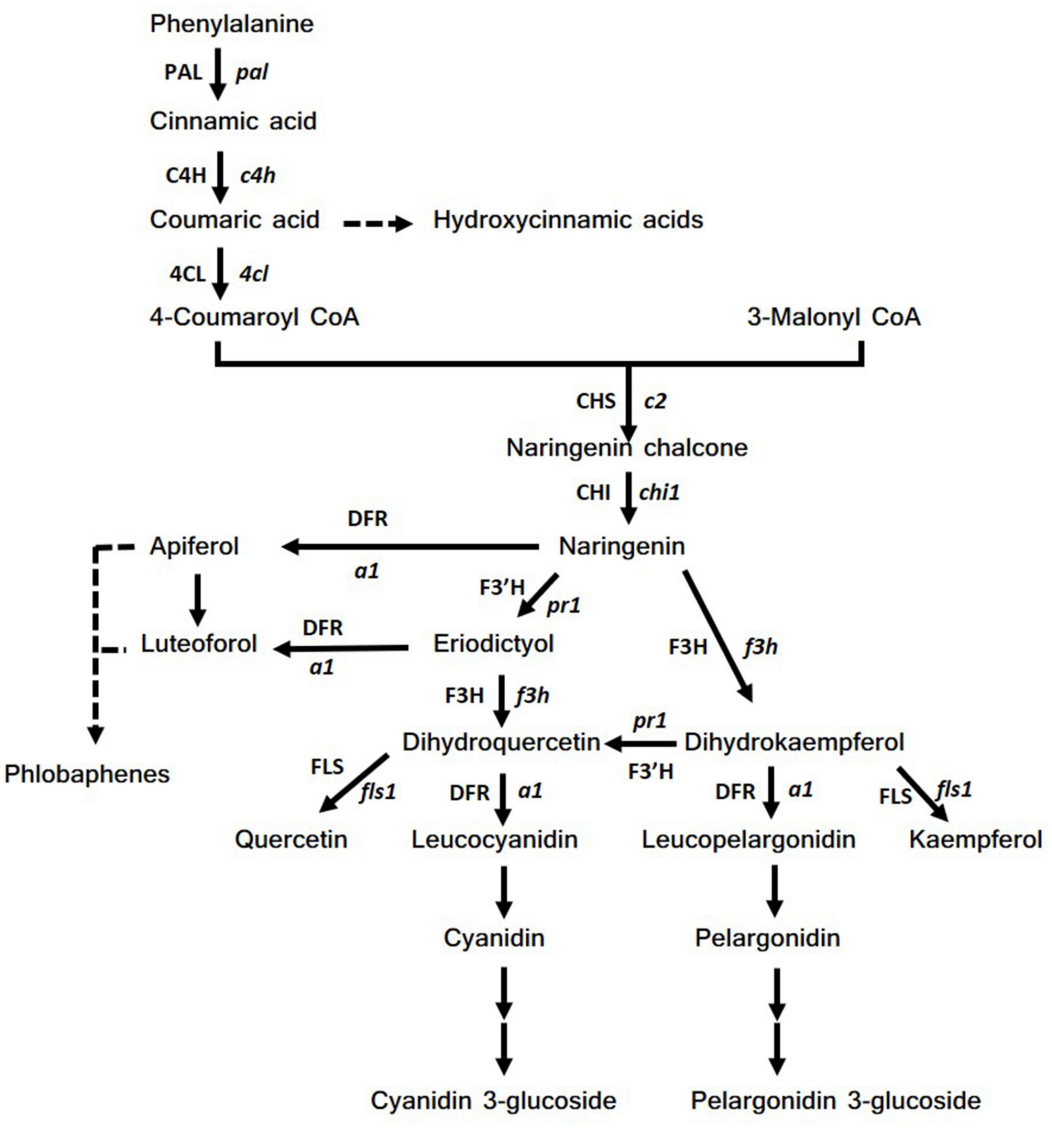
Scheme of flavonoids biosynthetic pathway. Abbreviations of genes (enzymes): pal (PAL), phenylalanine ammonia lyase; c4h (C4H), cinnamic acid 4-hydroxylase; 4cl (4CL), 4-coumarate-CoA ligase; c2 (CHS), chalcone synthase; chi1 (CHI), chalcone isomerase; f3h (F3H), flavanone 3 hydroxylase; pr1 (F3′H), flavonone 3'-hydroxylase; a1 (DFR), dihydroflavonol synthase.

The pathway starts from the condensation of four molecules of coumaroyl-CoA with 3-malonyl-CoA, which produces naringenin chalcone by the enzyme chalcone synthase, CHS (Figure 2). Naringenin chalcone is subsequently isomerized by chalcone isomerase (CHI) to naringenin, a key intermediate of the biosynthetic pathway. Naringenin is then transformed to dihydrokaempferol, which in turn is the substrate for three enzymes: (1) dihydroflavonol reductase (DFR) which leads to pelargonidin synthesis; (2) flavonol synthase (FLS) that transforms dihydrokaempferol to kaempferol; (3) flavanone 3-hydroxylase (F3′H) that catalyzes the formation of dihydroquercetin. Similarly, dihydroquercetin leads to the synthesis of cyanidin by dihydroflavonol reductase (DFR) or to the formation of quercetin by FLS. F3′H is also a key enzyme for the synthesis of phlobaphenes (41). These red pigments are formed from polymers of luteoforol and apiferol, which in turn derive from eriodictyol and naringenin, through the action of the DFR enzyme. Moreover, eriodictyol can be converted to dihydroquercetin by the activity of F3H (42). All these structural genes are regulated by the presence of two multigene families, c1/pl1/p1 genes, belonging to the family of MYB transcription factors and r1/b1 genes, belonging to MYC transcription factors (43–45). Usually, an active form of each family (acting as dominant) must be present to lead anthocyanin biosynthesis in different plant tissues according to the presence of different alleles.

There are various studies that highlight the wide range of biological activities of flavonoids, such as antiviral (46), antioxidant (47), anticancer (48), antimicrobial (49), and anti-inflammatory (50). As antivirals, several flavonoids have been reported to inhibit the targets of SARS and MERS coronaviruses (51) in different ways: blocking the enzymatic activities of viral proteases (3CLpro and PLpro), interfering with spike glycoproteins or suppressing the activity of ACE2 receptors (52, 53), which not only play an important role in cardiovascular diseases, but can be a key factor in viral infections and pneumonia (54). In particular, the hydroxyl group of flavonoids at 7-position appears essential to attack the binding site against 3CLpro and PLpro.

Different studies have focused primarily on the interference of flavonoids with the main viral proteases of SARS and MERS coronaviruses by using tools such as common enzymatic activity measurement, FRET (fluorescence resonance energy transfer) based methods and molecular docking (55–57). 3CLpro and PLpro are both key targets as they process many viral polyproteins that are involved in RNA replication and transcription within host cells (58).

However, the majority of the studies dealing with the health beneficial properties of flavonoids are conducted *in vitro* on the basis that these compounds show poor stability, low bioavailability, and poor distribution when tested *in vivo* (52). Among the tools and strategies used to increase these functions, the most promising are the insertion of structural modifications of the molecules (59), the use of absorption enhancers and nanotechnology (60, 61).

In the next step, epidemiological work should focus on clinical trials on COVID-19 patients in order to point out a reduction of the virus multiplication in the patient's body and a decrease in clinical signs (62). Furthermore, the advantage of implementing the diet with flavonoids is related to their high safety profile and lack of major side effects (62).

In this context, several authors have recently suggested kaempferol, quercetin, naringenin, curcumin, catechin, and epicatechin-gallate as recommended compounds found in plants that may act against COVID-19 proteases (18, 19, 26).

### Quercetin

Quercetin is one of the most important flavonoids and belongs to the class of flavonols (Table 1). Quercetin is the aglycone form of several glycoside flavonoids: rutin and quercitin are the most common. In fact, sugars such as glucose, rhamnose, galactose, and rutinose are usually bound to these natural compounds to form glycosides. Quercetin is naturally present in several fruits and vegetables, and also in medicinal herbs (63, 64). Its highest concentration is present in capers (*Capparis spinosa* L.), which contain 234 mg of flavonol per 100 g of edible portion. Due to its beneficial properties, quercetin is used as a food supplement and can counteract various diseases, acting as antiviral, anticancer, antioxidant, antidiabetic, antiulcer, antiallergy, antihypertensive, anti-inflammatory, and was reported to protect the human body from cardiovascular and gastrointestinal diseases (65). Recently, Solnier et al. have proposed quercetin as a good anti-SARS-CoV-2 candidate (19). In fact, several flavonols showed antiviral activity against coronaviruses (such as SARS-Cov and MERS-CoV) through the inhibition of 3CL and PLpro proteases (51). Since the former SARS-CoV and the new SARS-CoV-2 show high sequence similarity in the spike glycoproteins, flavonols may be also expected to prevent the entry of SARS-CoV-2 into host cells. Moreover, it has been demonstrated that the spike protein of the novel virus binds the ACE2 receptor with higher affinity compared to SARS-CoV (58). Therefore, the inhibition of ACE2 through a competing binding, appears to be a good approach to prevent SARS-CoV-2 infections. In this framework, experimental results have demonstrated that quercetin exerts strong inhibitory effects on ACE2 *in vitro*, and also *in vivo* when tested in rats (52–54, 66). Furthermore, the screening of a library of 150 compounds, allowed the identification of quercetin as a potent inhibitor of SARS-CoV-2 3CLpro (67). Taken together, these results suggest that quercetin may prevent the entry of SARS-CoV-2 in the host cell, binding the S protein and inhibiting ACE2 receptors.

### Kaempferol

Another important flavonol is kaempferol (Table 1), a secondary metabolite found in a wide variety of edible plants and food-derived products (24), such as kale, common bean, cabbage, broccoli, endive, and leek. The highest level of this compound was found in capers and saffron (259 and 205 mg/100 g, respectively). The glycoside form of kaempferol is astragalin, well-known for its multiple therapeutic properties (68) such as antioxidant (69, 70), anti-inflammatory (71), anticancer (72), neuroprotective (73), and antiviral (25).

### Naringenin

A natural compound that belongs to the flavanone class is naringenin (Table 1). Naringenin is present in a wide variety of fruits and vegetables, but the highest concentrations are reported in grapefruit, tangerines, oranges, and tomatoes. Naringenin mainly occurs as glycosides such as naringin or prunin (74). The presence of this flavanone in human diet is relatively high (75), but its bioavailability is limited (nearly 5.81%). Moreover, it appears that the glycosylated form naringin is less bioavailable than the respective aglycone (76). However, in order to solve the problem of naringenin's limited bioavailability, some formulations such as nanoparticles loaded with naringenin have been developed (77). After the absorption via active transport and passive diffusion (78), naringenin attaches to albumin and is finally transported to different organs: brain, liver, kidneys, and heart (79). Like other flavonoids, naringenin was found to be endowed with beneficial strong antioxidant, anti-inflammatory and antiviral properties (80–82). In particular, the antioxidant role of this flavanone was shown to be carried out by eliminating free radicals and preventing DNA oxidative damage (83–85) while the strong anti-inflammatory activity is due to the inhibition of the NF-kB (nuclear factor kappa B) signaling pathway (86) since NF-kB promotes the expression of many fundamental inflammatory proteins (87). The antiviral activity of naringenin was tested against some viruses: HCV, Dengue virus (DENV), Chikungunya virus (CHIKV), and Zika virus (ZIKV) (26). In this context, the beneficial properties and the possible therapeutic effects of naringenin against SARS-CoV-2 have been recently reviewed (26), pointing out that it may exert therapeutic effects against COVID-19 through the inhibition of the main protease 3CLpro and the reduction of ACE2 activity. Moreover, one additional mechanism by which this flavanone can counteract the effects of SARS-CoV-2 infection can be attributed to the attenuation of inflammatory responses.

### Hesperetin

Another flavonone similar to naringenin is hesperetin (Table 1), mainly found in the glycoside form (hesperidin) in citrus fruits, where it is particularly abundant in the peel and in the white part of the fruit. Therefore, the consumption of the whole fruit would ensure a greater intake than the juice alone (88, 89). As recently reviewed (90), the content of hesperidin for 100 mL of juice varies according to the fruit: in oranges it ranges from 20 to 60 mg, in lemons from 4 to 41 mg, in tangerines the content is between 8 and 46 mg, while in grapefruit it is lower (2–17 mg). Among all flavonoids, researchers have recently focused the attention on hesperidin because the low binding energy of hesperidin to the spike glycoprotein and to the protease 3CLpro suggests an effective antiviral action (29). In addition, hesperidin is considered an important antioxidant compound (29), able to counteract the damaging effects of oxygen free radicals, triggered by infection and inflammation.

## Other Aromatic Compounds

### Curcumin

Curcumin is a natural phenolic compound found in turmeric (*Curcuma longa* L.), a plant native to India and Southeast Asia where curcumin is used as a traditional medicine to treat various disorders. In Europe, this molecule is used as a food dye for its yellow color and it is classified as a food additive. Curcumin is characterized by multiple beneficial properties, acting as anti-inflammatory, antineoplastic, antiangiogenic, but also as an antiviral (influenza virus, hepatitis C virus, HIV), antibacterial (*Streptococcus* spp., *Staphylococcus* spp. and *Pseudomonas* spp.) and antifungal (*Candida* spp., *Aspergillus* spp., *Cryptococcus* spp., and *Dermatophytes* spp.) natural compound (91). It is active against various human viruses, bacteria and fungi. Nowadays, foods with high curcumin content have been evaluated as SARS-CoV-2 inhibitors (Table 1). Despite its poor bioavailability, some nanoparticle-based approaches have recently been developed (92–94). Furthermore, it was shown that different compounds can increase curcumin bioavailability. In particular, when combined with piperine, the major active component of black pepper, curcumin can increase its bioavailability as much as 20-fold (95).

### Phloretin

Another natural phenol is phloretin (Table 1). Phloretin is a dihydrochalcone and phlorizin is its main glucoside. Both compounds are naturally present in apples, kumquat, pear, strawberry, and vegetables (96, 97). Phloretin is a flexible molecule able to efficiently bind biological macromolecules. It is endowed with antiviral as well as anticancer, antifungal, anti-inflammatory, and antibacterial properties, thus conferring important health-beneficial effects (32). Furthermore, this compound can increase the fluidity of membranes and enhance the penetration of administered drugs into cells (98, 99).

### EGCG

An additional compound under research for its beneficial properties on human health which may be interesting under the antiviral activity aspect is epigallocatechin gallate (EGCG) (Table 1). EGCG is a type of catechin and it is abundant in green tea (100), while small quantities are also present in onions, plums, and apple skin. EGCG is a strong antioxidant and antitumor molecule and has the potential to prevent and counteract several human diseases with chronic metabolic and inflammatory components, such as diabetes, stroke, obesity, Parkinson's, and Alzheimer's diseases (101–103). Probably due to its ability to interact with DNA methyltransferases (DNMT), ACE-2 and helicase, EPGCG is also an antiviral molecule able to counteract diseases caused by a wide variety of viruses: herpes simplex virus (HSV), human papillomavirus (HPV), adenovirus, hepatitis B and C viruses (HBV and HCV, respectively), dengue virus (DENV), Zika virus (ZIKV), West Nile viruses (WNV), Chikungunya virus (CHIKV), Ebola virus (EBOV), human immunodeficiency virus (HIV), and influenza virus (104–108).

## Sulfur Compounds

### Sulforaphanes

Sulforaphanes are not phenolic compounds, but possess antiviral potential. They belong to the isothiocyanate group of nitrogen-containing plant secondary metabolites and are classified as sulfur compounds (Table 1). Sulforaphanes are stored as glucoraphanin, their inactive form (109). This natural compound is principally found in cruciferous vegetables (such as broccoli), is used in prevention and support of chronic diseases and is supposed to be involved in human aging (110). Moreover, it has been suggested that sulforaphane, like other natural phytochemicals, may be used in SARS-CoV-2 treatment (36). Cruciferous plants are able to release glucoraphanin, converted by the plant into sulforaphane, which in turn activates Nrf2 (111), an important transcription factor that induces an antiviral action and prevents oxidative stress (112). Nrf2 activity decreases with age, causing the elderly to be more susceptible to oxidative stress-mediated diseases (36).

## Strategies to Obtain Flavonol Rich Foods

**Figure 3:**
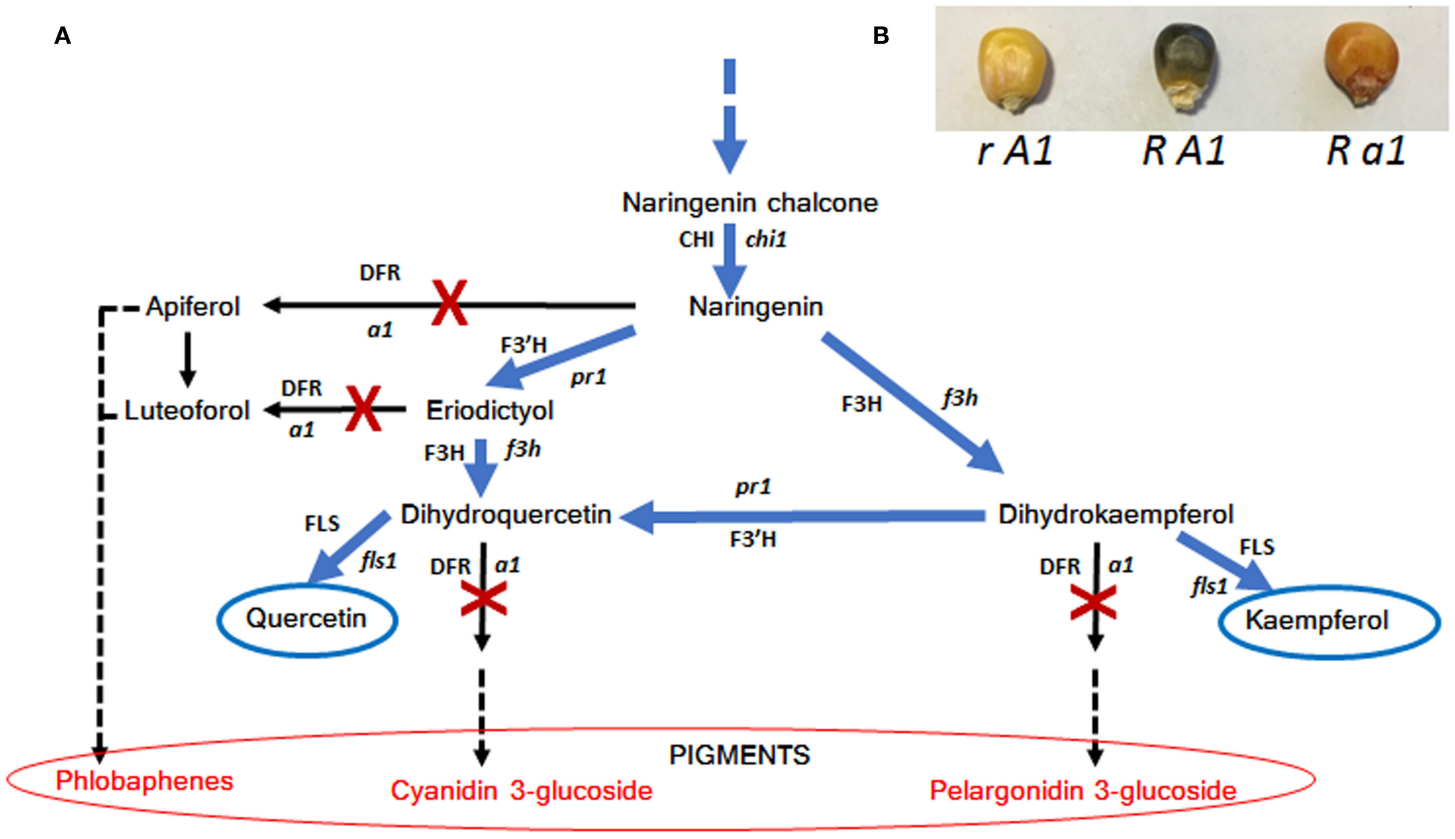
Schematic representation of how plants accumulate quercetin and kaempferol by stopping the activity of DFR **(A)** and **(B)** effect of a1 (DFR) mutation on maize seed pigmentation. Genes (enzymes) are abbreviated as follows: c2 (CHS), chalcone synthase; chi1 (CHI), chalcone isomerase; f3h (F3H), flavanone 3-hydroxylase; pr1 (F3′H), flavonone 3′-hydroxylase; a1 (DFR), dihydroflavonol synthase. In **(B)** R represent red color 1, a regulatory gene conferring anthocyanin pigmentation in the aleurone seed layer.

To our knowledge, caper (*Capparis spinosa* L.) is one of the edible species capable of accumulating the highest levels of quercetin (quercetin-3-rutinoside, named rutin), a flavonol compound with various curative properties (113, 114). Another plant capable of accumulating high levels of rutin in the seed is buckwheat (Fagopyrum spp.) and in particular *Fagopyrum tataricum* Gaertn that, compared to *Fagopyrum esculentum* Moench, is able to accumulate 40–50 × higher amounts of rutin (115, 116). In both cases this flavonol is synthesized via the flavonoid biosynthetic pathways where the main genes are PAL, C4H, 4CL, CHS, CHI, F3′H, F3H, FLS, and UFGT (Figure 2); these genes are very highly conserved among different plants and after the first characterization in maize they were studied in different species such as Arabidopsis, petunia, snapdragon and buckwheat (42, 117, 118). Therefore, it seems that the ability to accumulate large quantities of quercetin is determined by the inactivity of a key gene for anthocyanin biosynthesis, the DFR gene. In fact, the activity of DFR would lead to the synthesis of anthocyanins and/or phlobaphenes by subtracting the common precursor naringenin (Figure 2). As a consequence, both caper and buckwheat are not able to accumulate large amounts of anthocyanins in their tissues, as can also be observed for example in flowers that have colorless or weakly pigmented petals. To strengthen this hypothesis, the mutation in *anthocyaninless1 a1* (DFR) maize gene, in a genetic background prone to anthocyanin synthesis, has been reported to cause suppression of anthocyanin production followed by an accumulation of quercetin in the aleurone layer conferring a brownish color (119) (Figure 3). Hence the strategies that can be used to increase the flavanone content in food can be summarized as below:

1. the rediscovery of traditional varieties (landraces) naturally rich in these molecules.
2. the use of classical breeding techniques to specifically drive the accumulation of these molecules.
3. a biotechnological approach such as CRISPR/Cas9 to inactivate the DFR gene in pigmented varieties.

All these methods can be used in synergy to increase the flavonol content in foods.

The species/varieties rich in flavanones such as quercetin or naringenin will be described in the next paragraph. The strategy 1 is the simplest and most user-friendly since it is based on the selection of pre-existing varieties, while the second strategy, concerning breeding techniques, takes several years, and the third, based on the novel NBTs (new breeding techniques), currently has to comply with the same European regulations as those for GMOs (120).

Considering corn as a case study, both traditional and new corn varieties obtained by breeding (121–123) are rich in flavonoids, mainly anthocyanins and phlobaphenes [reviewed by (42, 124, 125)]. These varieties, in addition to being rich in anthocyanin pigments, are able to accumulate discrete quantities of flavonols as previously reported by Lago et al. (124), Cassani et al. (125), and Landoni et al. (126). However, in order to further increase significantly the content of flavonols (in particular quercetin), it would be enough to introduce the a1 mutation by recurrent backcrossing.

## Varieties Rich in Flavonoids

Many cultivated plants are rich in bioactive compounds and mineral elements with potential health benefits (127). A strong variability in the flavonoid content is present among different species and varieties. In Supplementary Table 1 we present different cultivars of fruits and vegetables highlighting the different contents of quercetin, kaempferol, hesperidin and naringenin which have been found in them.

Regarding quercetin, as already mentioned, capers are the richest food, reaching 2,340 mg/kg (20). Onions are also a good source of quercetin: white varieties had a level of 900–1,830 mg/kg, while the red variety Karmen had a content of over 2,500 mg/kg (128). Although pink onions were also rather rich in this compound, the registered level was lower compared to the others. Quercetin is also present in lovage and dill, two aromatic herbs widely used in cooking (129). In Bronte pistachios, quercetin is mainly contained in the skin, so the availability during consumption is relatively low, considering that the peel is only 10% of the whole nut (130). A comparison of the phenolic profiles from available literature data about radicchio *(Cichorium intybus)* cultivars allowed the selection of three autochthonous varieties *(“Verdon da Cortèl,” “Treviso Precoce,” “Chioggia”*) cultivated in the Veneto region of Italy. The variability between cultivars ranged from 40 to 250 mg/kg) (131). The most abundant forms of quercetin present in radicchio are quercetin glucuronide (3-O; 7-O) and quercetin-3-O-glucoside. Furthermore, the glycoside form of quercetin (rutin, quercetin-3-O-rutinoside) is present in buckwheat and asparagus. The selected varieties of buckwheat contained rutin in concentrations ranging from 590 to 769 mg/kg (116). A great intraspecific variability was observed in asparagus: the highest rutin values were recorded in green cultivars (119–163 mg/kg), while the lowest in the white (2 mg/kg). Purple varieties had intermediate levels of rutin (15–20 mg/kg) (132).

As to the other flavonol, kaempferol, it is present in its aglycone form in saffron (2,050 mg/kg) (133) and in its glycosylated forms in capers and radicchio (23, 131, 134).

The flavonones hesperetin and naringenin are present in citrus fruits in their glycosylated forms hesperidin and naringin (Supplementary Table 1). They are mainly found in the peel and albedo (white part) of citrus fruits at higher values compared to the juice. In fact, the consumption of fresh fruit allows a greater intake (29). The highest hesperidin levels in 100 mL of juice were recorded in oranges (20–60 mg), but also in mandarins and clementines (8–46 mg) (29). According to Alam et al., the concentration of naringenin in 100 mL of mandarin juice was 300 mg (135). The concentration of this compound in mandarins was 10 times higher compared to grapefruit and over a hundred times higher than that of oranges. In tomatoes, three cultivars appeared to have the highest naringin concentrations: Daniella 12.55 mg/kg, Ramillete 8.14 mg/kg and Canario 8.46 mg/kg. Moreover, these varieties also appeared to have also the highest values of the flavonol quercetin (136).

## Dishes Rich in Flavonoids: Bioavailability and Cooking Processes

Establishing the bioavailability of bioactive compounds is a fundamental step in determining the effects of phytochemicals on human health (137). From a nutritional point of view, bioavailability is defined as the fraction of a given molecule contained in food that the body can utilize (138, 139). Bioavailability is the result of different processes: digestion, absorption, metabolism and elimination of a compound after food ingestion (137). Flavonoids are first metabolized by phase I and phase II metabolism which take place in the gastrointestinal tract and liver, and subsequently by microbial metabolism in the colon (140). Finally, urinary excretion and plasma concentrations of flavonols in humans could be used in epidemiological studies as biomarkers of intake (141). However, the bioavailability of these compounds varies between individuals and many factors such as age, sex, genotype, but also food composition may affect these metabolic processes (140).

Although some vegetables mentioned in Supplementary Table 1 can be used as fresh products, the majority of flavonoid rich foods require a cooking process before being consumed (142). Such a process can modify the chemical-physical properties of any phytochemical as well as its bioavailability. The first losses of flavonoids may occur in the pre-cooking stages in cases where parts of the product are removed. Onion peeling resulted in a 39% loss of flavonoids and asparagus chopping produced an 18.5% decrease of the rutin content (143, 144). Considerable losses were also recorded in the peeling and dicing of tomatoes (145). Thermal processes (blanching, drying, pasteurization, microwaving etc) also have an impact on the flavonoid content which depends on the magnitude and duration of heating (142). The data reported by Ioannou and Ghoul revealed a different sensitivity to heat treatment for the different flavonoids in aqueous solution. In general, a significant degradation was observed for temperatures above 100°C (142). A higher stability compared to the aglycon form was however observed in rutin (146, 147). During boiling, a fraction of the flavonoids is released into the cooking water causing losses of 20.5% for onions and 43.9% for asparagus (144). Furthermore, onion frying processes diminish flavonoid content (25–33%) (148, 149), while microwaving and steaming do not have a significant effect (149, 150). In contrast, baking increases the total flavonol content in onions (7%) (150). The degradation of flavonoids depends also on other factors such as pH and the presence or absence of oxygen. In fact, the presence of oxygen accelerates the degradation of rutin and quercetin due to the formation of ROS (146, 147).

Ioannou et al. showed the effects of temperature, oxygen and light on naringin content (151). This compound is degraded at temperatures above 100°C, with an oxygen content over 85% or upon exposure to light. In fact, a decrease in the naringin content was recorded by applying 108°C (400 W microwave power). However, by setting the extraction temperature at 80°C, an increase in naringin was observed (151).

A mixture of flavonoids-rich foods is present in the sofrito, widely used in the Mediterranean diet (152). Sofrito is composed of several ingredients rich in phenolic compounds, such as tomatoes, onions and olive oil (153). Naringenin is the main flavonone present in fresh tomatoes and tomato sauces (154, 155) and its content in sofrito is higher compared to tomatoes. By adding 120 g of sofrito to different dishes (such as pasta), a phenolic compound intake of 15–25 mg occurs (152). Adding capers to the sofrito could be a good strategy to increase the content of quercetin and kaempferol. If we consider the possible daily intake of 20 capers (10 g), this will provide from 20 to 80 mg of quercetin (23).

In this context, the varieties richest in these bioactive natural compounds can be implemented in human diet in order to try to protect to some extent from COVID-19.

Considering only quercetin, one of the most abundant dietary flavonoids with a daily average intake of 25–50 g (156), several papers cited in the flavonoid section of this review claimed its physiological effects on inflammation and immune function in murine systems [reviewed by Li et al., (157)]. These effects are linked to a daily intake ranging from 10 to 160 mg per kg. In particular, considering the effect on pro-inflammatory and anti-inflammatory cytokines production, linked to the response to virus infection, the dosage effective on the rat system was reported to be about 10–20 mg/kg (158). From these data we could infer a recommended human daily intake of about 500 mg, considering an average body weight of 65 kg. Several dishes rich in quercetin could fully satisfy this recommended daily intake, with a possible positive effect on human health (Table 2).

**Table 2:** Dishes rich in quercetin.

**Dish**	**Ingredients**	**Indicative quantity (g/serving)**	**Quercetin content (mg/100 g)**	**Quercetin content (mg/serving)**	**Processing**	**Quercetin loss (%)**	**Quercetin intake after processing and cooking (mg/serving)**	**Quercetin intake after processing and cooking) (% RDA)**	**References**
Salad	Radicchio (cv. Chioggia)	100	17	17	Fresh	–	17	0.34	(143)
	Onion (cv. Karmen)	40	254.9	102	Peeling, trimming	39%	62.2	12.44	
	Tomato (cv. Daniella)	100	43.6	43.6	Fresh	–	43.6	8.72	
	Capers	10	234	23.4	Fresh	–	23.4	4.68	
	Olive oil	q.s	–	–	–	–	–	–	
	Salt	q.s	–	–	–	–	–	–	
TOT			549.5	186	–	–	146.2	26.18	
Pasta with tomato sauce	Tomato sauce (cv. Daniella)	200	43.6	87.2	–	–	87.2	17.44	(159)
	Onion (cv. Karmen)	40	254.9	102	Peeling, trimming	39%	77.8	15.56	(143)
	Capers	10	234	23.4	Fresh	–	23.4	4.68	
	Pasta	80	–	–	–	–	–	–	
	Olive Oil	q.s	–	–	–	–	–	–	
	Salt	q.s	–	–	–	–	–	–	
TOT			488.9	125.4	–	–	101.2	37.68	
Polenta taragna	Buckwheat flour (cv. Valtellinese)	50	764	382	–	29%	271.2	54.24	(160)
	Corn flour	20	–	–	–	–	–	–	
	Water	300	–	–	–	–	–	–	
	Salt	q.s	–	–	–	–	–	–	
TOT			764	382			271.2	54.24	

*[RDA inferred from (158)]*.

## Conclusions and Future Perspectives

This survey of the properties of several phytochemicals present in edible organs of many cultivated plants appears to support the concept that people hit all over the world by the COVID-19 pandemic can rely on a very easily usable tool that may contribute to prevent the disease and/or decrease its severe effects which are causing so much lethality. This tool is simply the integration into our diet of the natural foods which selectively implement the daily intake of a few bioactive phytochemicals proven to possess properties which provide some degree of protection against COVID-19. Such natural compounds have in fact been shown to be able to put in place mechanisms of prevention and/or even inhibition of viral infection/replication. The plant derived molecules of anti-Covid interest belong mostly but not exclusively to the chemical class of the flavonoids. In particular, the flavonol quercetin and the flavanones naringenin and hesperidin appear the best candidates to play the role of anti-Covid shelters, particularly because of their ubiquitous spread in many edible fruit and vegetables of large consumption in which they can be found at high levels. Curcumin, a phenolic compound present in *Curcuma longa* roots is another phytochemical of interest because it is largely extracted and used as a food dye, so it can be easily integrated into the diet. Among other phenolics displaying antiviral activity, phloretin is also to be taken into account as an anti-Covid shelter because it is rather ubiquitous in vegetables and fruits (among them, apples and pears) and epigallocatechin gallate (present mostly in green tea, onion, plum, apple skin) because, besides being endowed with antiviral properties, it displays potential for the mitigation of diseases characterized by a chronic inflammatory component. Finally, the sulfur compounds sulforaphanes, diffused in cruciferous plants such as broccoli, may be antiviral shelters of particular interest because their action relies on the activation of transcription factors which in turn switches on cell mechanisms responsible for antiviral effects. The major future perspectives for enhancing and diffusing the above cited, already known and highly desirable plant-derived biochemical weapons to fight COVID-19, can be summarized in three different strategies and some examples of each of them are reported in the preceding paragraphs. The first one consists in the rather easily doable rediscovery of ancient varieties naturally rich in these molecules; as concerns this approach, it is known that traditional varieties/ecotypes are often richer in phytonutrients than the newly synthesized varieties where the improvement was mainly focused on yield (116, 125, 161). The second strategy is the use of classic genetic improvement techniques to enhance specific accumulation of a given antiviral phytochemical. The third is the use of biotechnological approaches, nowadays available and quite effective, such as CRISPR/Cas9, which are able to activate or, conversely, to inactivate genes involved in the synthesis of specific antiviral phytochemicals leading to the accumulation of specific compounds.

Moreover, in order to modulate and optimize the “functional diet,” it will be necessary to further increase information concerning the actual levels each phytochemical reaches in the blood following intake of food or of nutraceutical preparations endowed with anti-Covid potential. In this regard, in cases in which fruits or vegetables containing the anti-Covid phytochemical must be cooked to be consumed, it will be also necessary to investigate more accurately and extensively the fate of these molecules during the cooking process and determine their absorption rate and extent.

It is probable that in the next decades many other phytochemicals capable of fighting human viral diseases will be found in the edible parts of plants and thoroughly characterized, since much research is at present under way to achieve this goal. Indeed, it seems that nowadays there is an increasing tendency to prefer or juxtapose to the pharmaceutical therapies, preventive or (more rarely) curative treatments based on bioactive nutraceuticals extracted from plants. So, it does not seem impossible that in the future, whenever possible, many diseases will be fought more “naturally” through a more focused and specific education of people's diet.

## Author Contributions

RP, EN, and ML contributed to conception and design of the study. MG, FC, SS, and ML wrote the first draft of the manuscript. All authors contributed to manuscript revision, read, and approved the submitted version.

## Conflict of Interest

The authors declare that the research was conducted in the absence of any commercial or financial relationships that could be construed as a potential conflict of interest.
