# Supplementary material for: Food Containing Bioactive Flavonoids and Other Phenolic or Sulfur Phytochemicals With Antiviral Effect: Can We Design a Promising Diet Against COVID-19?
Source: Front Nutr. 2021 Jun 17;8:661331. doi: 10.3389/fnut.2021.661331 (PMC8247467; doi:10.3389/fnut.2021.661331)
Supplement: Supplementary file 1 [file Table_1.DOCX]

| Sup. Mat. Table 1. Plants varieties richest in quercetin, kaempferol, hesperetin and naringenin. | | | | | | |
| --- | --- | --- | --- | --- | --- | --- |
| Aglycones | **Glycosylated form** | **Main sources** | **Cultivar** | | **Concentration *(mg/kg)*** | **Literature** |
| Quercetin | total glycosylated forms | Caper  (*Capparis spinosa*) | - | | 2340 | Derosa et al., 2020;  Giuffrida et al., 2002 |
|  |  | Onion (*Allium cepa*) | Red | Karmen | 2549 | Slimestad et al., 2007 |
|  |  |  |  | Morada de Amposta | 943 |  |
|  |  |  |  | Tropea rossa | 763 |  |
|  |  |  | Pink | Rose | 719 |  |
|  |  |  | Yellow | Vsetana | 1831 |  |
|  |  |  |  | MSU4535 | 1285 |  |
|  |  |  |  | Dorata Density | 979 |  |
|  |  | Lovage  (*Levisticum officinale*) | - | | 1700  (fresh weight) | Justesen et al., 2001 |
|  |  | Dill  (*Anethum graveolens*) | - | | 480-1100 (fresh weight) |  |
|  |  | Pistacia  (*Pistacia vera*) | Bronte | | 230 | Tomaino et al., 2010 |
|  |  | Tomato  (*Lycopersicum esculentum*) | Daniella | | 43.59 | Martínez‐Valverde et al., 2002 |
|  |  |  | Ramillete | | 28.66 |  |
|  |  |  | Canario | | 28.08 |  |
|  |  | Sweet bell pepper  (*Capsicum annum*) | Green | | 26.5 | Sun et al., 2007 |
|  |  |  | Yellow | | 29.6 |  |
|  |  |  | Orange | | 29 |  |
|  |  |  | Red | | 34 |  |

| Aglycones | Glycosylated form | Main sources | | Cultivar | | Concentration *(mg/kg)* | Literature |
| --- | --- | --- | --- | --- | --- | --- | --- |
| Quercetin | Quercetin glucuronide (3-O; 7-O) | Radicchio  (*Cichorium intybus*) | | Verdon da Cortèl | | 268.8 | Tardugno et al., 2018 |
|  |  |  |  | Chioggia | | 169.6 |  |
|  |  |  |  | Treviso Precoce | | 169.2 |  |
|  | Quercetin-3-O-glucoside | Radicchio  (*Cichorium intybus*) | | Verdon da Cortèl | | 168.3 | Tardugno et al., 2018 |
|  |  |  |  | Treviso Precoce | | 103.8 |  |
|  |  |  |  | Chioggia | | 73.4 |  |
|  | Rutin (quercetin-3-O-rutinoside) | Buckwheat  (*Fagopyrum tataricum*) | | Cinese | | 769 | Giupponi et al., 2019 |
|  |  |  |  | Valtellinese | | 764 |  |
|  |  |  |  | Di Bolzano | | 590 |  |
|  |  | Asparagus (*Asparagus officinalis*) | | Green | Grolim | 163.19 | Kulczyński et al., 2016 |
|  |  |  |  |  | Eposs | 140.62 |  |
|  |  |  |  |  | Schwetzinger Meisterschuss | 119.43 |  |
|  |  |  |  | Purple | Huchel’s Alpha | 19.73 |  |
|  |  |  |  |  | Schwetzinger Meisterschuss | 15.44 |  |
|  |  |  |  |  | Eposs | 15 |  |
|  |  |  |  | White | Huchel’s Alpha | 2.3 |  |
|  |  |  |  |  | Eposs | 2.2 |  |
|  |  |  |  |  | Gijnlim | 2.2 |  |
| Kaempferol | Kaempferol | | Saffron  (*Crocus sativus*) | - | | 2050 | Carmona et al., 2007 |
|  | Kaempferol 3-rutinoside | | Caper  (*Capparis spinosa*) | - | | 2000 | Giuffrida et al., 2002;  Inocencio et al., 2000 |
|  | Kaempferol-3-O-glucoside | | Radicchio  (*Cichorium intybus*) | Rosa di Verona | | 675.5 | Tardugno et al., 2018 |
|  |  |  |  | Verdon da Cortèl | | 237.1 |  |
|  | Kaempferol-3-O-(6″-O-malonyl) – glucoside | | Radicchio  (*Cichorium intybus*) | Rosa di Verona | | 1348.8 |  |
|  |  |  |  | Verdon da Cortèl | | 123.7 |  |
| Aglycones | **Glycosylated form** | **Main sources** | | **Cultivar** | | **Concentration *(mg/100mL juice)*** | **Literature** |
| Hesperetin | Hesperidin  (Hesperetin 7-rutinoside) | | Citrus (C.) species | Red orange  *(C. sinensis)* | | 43.6 | Bellavite and Donzelli, 2020 |
|  |  |  |  | Clementine  *(C. clementine)* | | 39.9 |  |
|  |  |  |  | Sweet orange  *(C. sinensis)* | | 28.6 |  |
|  |  |  |  | Mandarin  *(C. reticulata)* | | 24.3 |  |
|  |  |  |  | Lemon  *(C. limon)* | | 20.5 |  |
|  |  |  |  | Lime  *(C. aurantifolia)* | | 1.8 |  |
|  |  |  |  | Grapefruit  *(C. paradisi)* | | 0.9 |  |
| Naringenin | Naringin  (Naringenin 7-O-glycoside) | Citrus (C.) species | | Mandarin  *(C. reticulata)* | | 338.36 | Alam et al., 2014 |
|  |  |  |  | Grapefruit  *(C. paradisi)* | | 23.0 |  |
|  |  |  |  | Bergamot orange  *(C. bergamia)* | | 2.23 |  |
|  |  |  |  | Sweet orange  *(C. sinensis)* | | 2.13 |  |
|  |  |  |  | Lime  *(C. aurantifolia)* | | 1.97 |  |
|  |  |  |  | Clementine  *(C. clementine)* | | 0.8 |  |
|  | Naringin | Tomato *(Lycopersicum esculentum)* | | Daniella | | 12.55 | Martínez‐Valverde et al., 2002 |
|  |  |  |  | Ramillete | | 8.14 |  |
|  |  |  |  | Canario | | 8.46 |  |
